# Longitudinal 16S rRNA Sequencing Reveals Relationships among Alterations of Gut Microbiota and Nonalcoholic Fatty Liver Disease Progression in Mice

**DOI:** 10.1128/spectrum.00047-22

**Published:** 2022-06-01

**Authors:** Aoxiang Zhuge, Shengjie Li, Pengcheng Lou, Wenrui Wu, Kaiceng Wang, Yin Yuan, Jiafeng Xia, Bo Li, Lanjuan Li

**Affiliations:** a State Key Laboratory for Diagnosis and Treatment of Infectious Diseases, National Clinical Research Center for Infectious Diseases, Collaborative Innovation Center for Diagnosis and Treatment of Infectious Diseases, The First Affiliated Hospital, Zhejiang University School of Medicine, Hangzhou, China; b Jinan Microecological Biomedicine Shandong Laboratory, Jinan, China; c Research Units of Infectious Disease and Microecology, Chinese Academy of Medical Sciences, Beijing, China; National Institutes of Health

**Keywords:** gut microbiota, NAFLD progression, *Akkermansia muciniphila*, fibrosis, bile acid metabolism

## Abstract

Nonalcoholic fatty liver disease (NAFLD) is a prevalent and progressive disease spectrum ranging from nonalcoholic fatty liver (NAFL) to nonalcoholic steatohepatitis (NASH), yet there is no effective treatment and efficient noninvasive diagnostic method for NASH. The present study investigated the longitudinal alternations of gut microbiota in the Western diet (WD) induced murine NAFLD model using 16S rRNA sequencing. Evident steatosis and inflammation were detected in the liver at the 8th and 12th week, while prompted hepatic oxidative injury and fibrosis were found at the 16th week. In this progressive process, impaired bile acid (BA) metabolism plays a vital part. Long-term WD intervention alters microbial richness and composition in the intestine, shaping characteristic microbial feature correspondence to each NAFLD stage. Descending abundances of *Clostridia* and *Ruminococcaceae* were found in NAFLD progression, while inflammation-related microbes *[Eubacterium]_fissicatena_group*, *Romboutsia*, and *Erysipelatoclostridium* were verified to identify borderline NASH at 8th and 12th week, and BA-associated taxa *Dubosiella*, *Bosea*, *Helicobacter*, and *Alistipes* were recognized as special symbols reflecting the state of oxidative damage and fibrosis in NASH at 16th week. Further, feces and colon abundances of *Akkermansia* were verified to be depleted in the process of borderline NASH progressed to NASH, and exhibited substantial correlations with NAFLD indexes ALT, AST, TC, and TBA. These characteristic taxa were effective to identify NAFLD and NASH, and microbiota-derived predictive models for NAFLD and NASH exhibited great potential (AUC 0.983 and 0.784). These findings demonstrate that a core set of gut microbiome especially BA-related taxa may be adopted as a noninvasive diagnostic tool for NAFLD and NASH.

**IMPORTANCE** This study concentrates on longitudinal alternations of gut microbiota in NAFLD progression and discovers the interrelationships between them. These findings may uncover the role of gut microbiota in NAFLD progression and identify novel noninvasive diagnostic tools for NAFLD based on microbial biomarkers.

## INTRODUCTION

Nonalcoholic fatty liver disease (NAFLD) is a common chronic liver disease with a global prevalence of 25% (1). NAFLD encompasses a progressive spectrum ranging from the nonalcoholic fatty liver (NAFL) with steatosis to nonalcoholic steatohepatitis (NASH), which is characterized by activated inflammation, developed fibrosis, and ultimately liver cirrhosis (1, 2). Instead of the outdated “two hits” hypothesis which attributes the development of NAFLD to initial steatosis and subsequent systemic inflammation, the novel “multiple parallel hits” hypothesis has proposed various pathogenic factors participating in NAFLD progression parallelly rather than consecutively, including insulin resistance, lipotoxicity, oxidative damage, endoplasmic reticulum (ER) stress, mitochondrial dysfunction, adipose tissue dysfunction, imbalanced innate immunity, cytokine secretion, and the gut-liver axis (3–6). Approximately 20% of cases of NASH will progress to liver cirrhosis and NASH is recognized to be the leading indication for liver transplantation (7). However, no pharmacological therapy targeting NAFLD has been approved only lifestyle change and weight reduction are suggested, which reflects the importance of early diagnosis for NASH (7–9). Except for liver biopsy specimens which served as the gold standard for NAFLD and NASH diagnosis, noninvasive diagnostic systems of NASH comprised of transient elastography, transaminase, cytokeratin 18 and other serum biomarkers have been explored, but none are widely accepted (7, 10–13). Thus, novel, biology-based, low-budget, easily accessible, highly sensitive, and specific noninvasive prognostic and diagnostic tools for NASH are urgently needed (14).

It has been demonstrated that gut microbiota is involved in the pathogenesis and development of NAFLD. Gut microbiota is capable of fermenting indigestible carbohydrates and yielding beneficial metabolites (for example, short-chain fatty acids and succinate), which work in the prevention and treatment of obesity and its comorbidities (15). Microbial dysbiosis will worsen gut permeability to bacterial products, aggravate bacterial translocation, and increase hepatic exposure to toxicants (endotoxins, ethanol, trimethylamine, etc.), causing dysmotility, gut inflammation, and other immunological changes to trigger hepatic inflammation and fibrosis (16). The gut microbiome also undergoes drastic changes in NAFLD patients. Oh et al. (17) indicated that Veillonella parvula, Veillonella atypica, Ruminococcus gnavus, Clostridium bolteae, and *Acidaminococcus* sp. *D21* are enriched in the NASH patients with fibrosis, in contrast to the depleted abundance of Eubacterium eligens, Eubacterium rectale, and Faecalibacterium prausnitzii. Considering these changes, using microbiota or microbiota-derived signatures (collected from feces or blood) as biomarkers may be a potential alternative for noninvasive diagnosis or as an auxiliary method for NASH (14).

However, the specific relationships between longitudinal alternations of gut microbiota and NAFLD profiles remain unclear. In this study, we established a progressive NAFLD model with Western diet (WD) feeding in mice recapitulating the human NAFLD clinical profiles, which are characterized by abnormal lipid accumulation, inflammation, oxidative damage, and activated bile acid metabolism and fibrosis. Subsequently, we utilized longitudinal 16S rRNA sequencing to track alternations in gut microbiota and investigate their interrelationships with NAFLD progression and found potential microbial biomarkers for noninvasive diagnosis of clinical NAFLD.

## RESULTS

### Western diet-induced progressive NAFLD features and liver injury.

**FIG 1:**
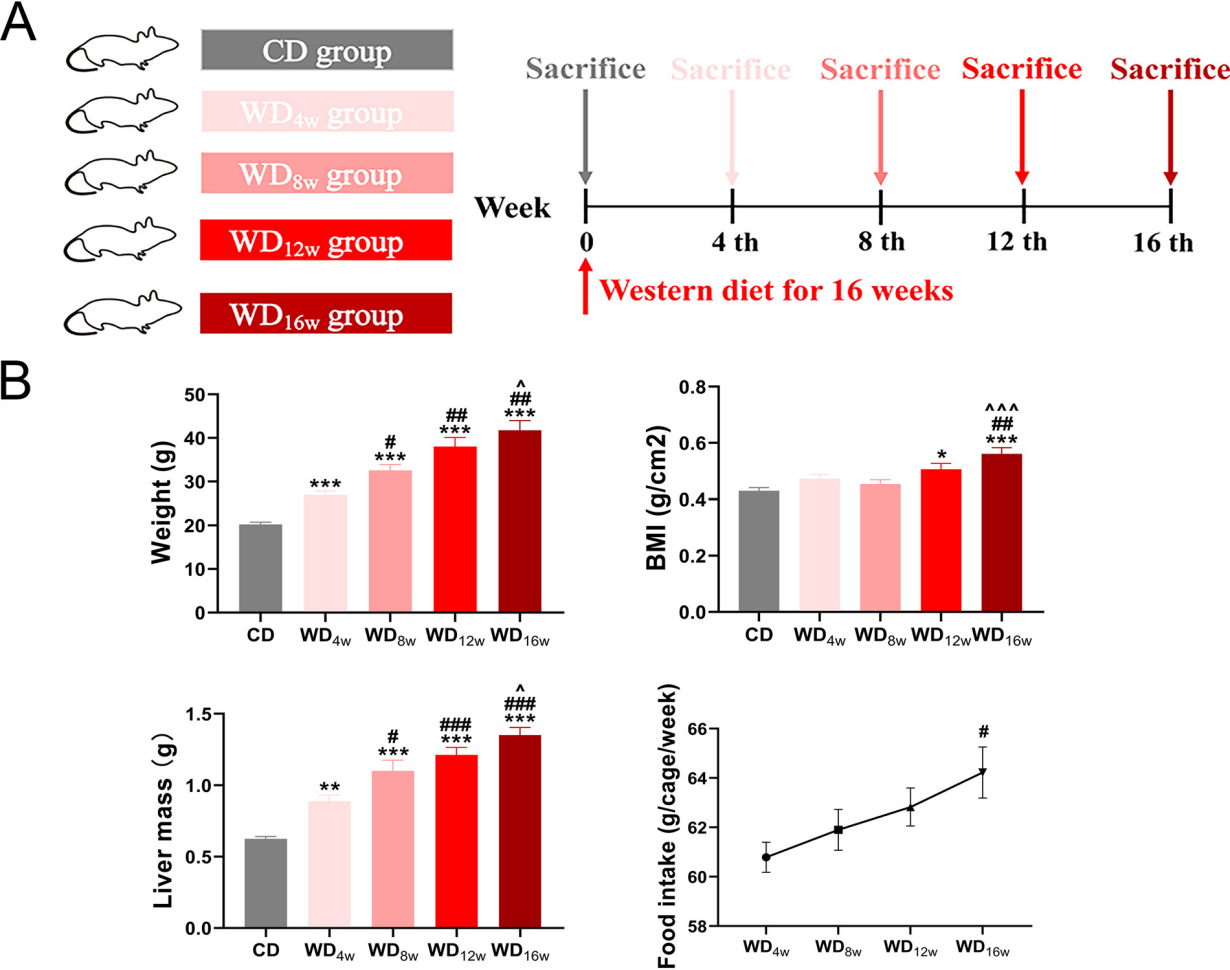
Effects of longitudinal western diet on weight and food intake change in mice. (A) Experimental design. (B) Changes in body weight, BMI, liver mass and food intake after Western diet feeding (*n* = 8 to 9 per group). Data are presented as mean ± SEM. * indicates a significant difference between the CD group and the other groups, *, *P* < 0.05; **, *P* < 0.01; ***, *P* < 0.001; # indicates significant difference between the WD_4w_ group and the other groups, ^#^, *P* < 0.05; ^##^, *P* < 0.01; ^###^, *P* < 0.001; ^ indicates significant difference between the WD_8w_ group and the other groups, ^^^, *P* < 0.05; ^^^^^, *P* < 0.001.

**FIG 2:**
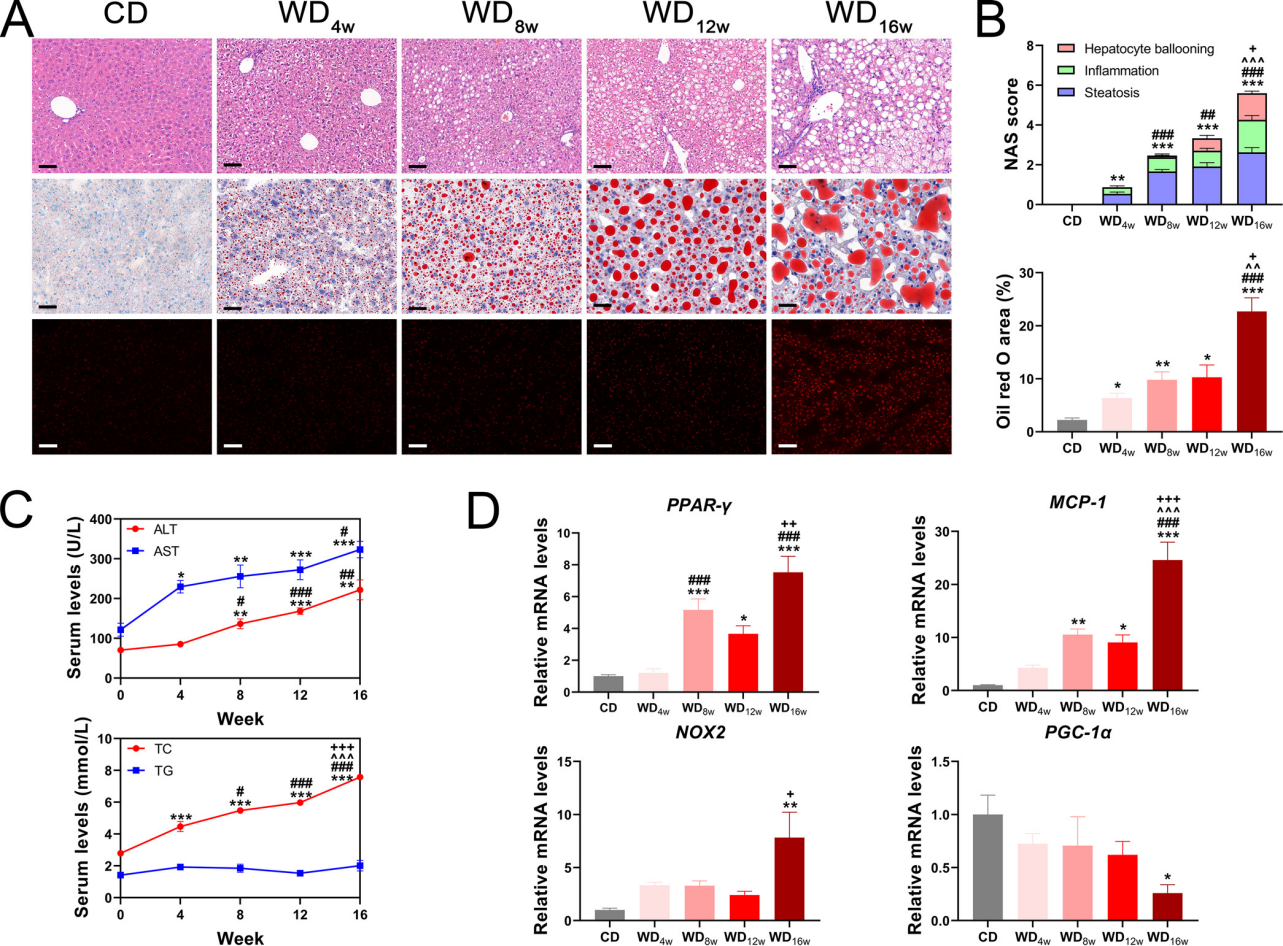
Longitudinal liver histopathology and liver injury of mice fed with a Western diet. (A) Representative images of the liver with H&E staining, Oil red O staining, and DHE immunofluorescence. Scale bar 20 μm. (B) NAS score and Oil red O area (*n* = 8 to 9 per group), (C) Seral levels of ALT, AST, TC, and TG (*n* = 8 to 9 per group), and (D) Liver expressions of *PPAR-γ*, *MCP-1*, *NOX2* and *PGC-1α* (*n* = 6 per group) at 0, 4th, 8th, 12th, and 16th week. Data are presented as mean ± SEM. * indicates a significant difference between the CD group and the other groups, *, *P* < 0.05; **, *P* < 0.01; ***, *P* < 0.001; # indicates significant difference between the WD_4w_ group and the other groups, ^#^, *P* < 0.05; ^##^, *P* < 0.01; ^###^, *P* < 0.001; ^ indicates significant difference between the WD_8w_ group and the other groups, ^^^^^, *P* < 0.001. + indicates significant difference between the WD_12w_ group and the other groups, ^+^, *P* < 0.05; ^++^, *P* < 0.01; ^+++^, *P* < 0.001.

NAFLD is a progressive disease spectrum that exhibits a series of distinctive histological features at different time points. However, there is not enough evidence to match each characteristic histological alternation to a specific time point. To address this concern, the developing NAFLD alternations at 0, 4th, 8th, 12th, and 16th weeks were investigated in WD-fed mice (Fig. 1A). As expected, WD significantly increased body weight, body mass index (BMI), and liver mass and displayed an upward trend in food intake along with the intervention (Fig. 1B). Further, hematoxylin and eosin (H&E) and Oil red O staining revealed progressively accumulated lipid droplets and excessive reactive oxygen species (ROS) in the liver (Fig. 2A and B). NAS score showed that NAFLD developed into borderline NASH at the 8th week and progressed to NASH at the 16th week (Fig. 2B). In line with the histological changes, alanine aminotransferase (ALT), aspartate aminotransferase (AST), and total cholesterol (TC) were also elevated, whereas triglyceride (TG) remained unchanged (Fig. 2C). Expressions of genes associated with lipid metabolism (*PPAR-γ*) and inflammation (*MCP-1*) were significantly upregulated at the 8th week, while oxidative damage markers (*NOX2* and *PGC-1α*) were unchanged until the 16th week (Fig. 2D). These results indicate that WD has long-term effects on NAFLD progression, which are characterized by apparent lipid accumulation, inflammation at the 8th week, and NASH with oxidative damage at the 16th week, respectively.

It has been proved that NAFLD progression is accompanied by gut barrier dysfunction (18), so we detected expressions of tight junction proteins *ZO-1*, *Occludin,* and mucus layer component *MUC2* in the ileum and found impaired gut barrier function as NAFLD progressed (Fig. S1A and B). To assess bacterial translocation induced by increased gut permeability, serum endotoxin levels were found elevated at the 16th week (Fig. S1C). Further, insulin resistance examination of mice in the WD_16w_ group demonstrated that long-term WD worse glucose tolerance and insulin resistance (Fig. S1D).

### Western diet-induced progressive NAFLD fibrosis under activated bile acid metabolism.

**FIG 3:**
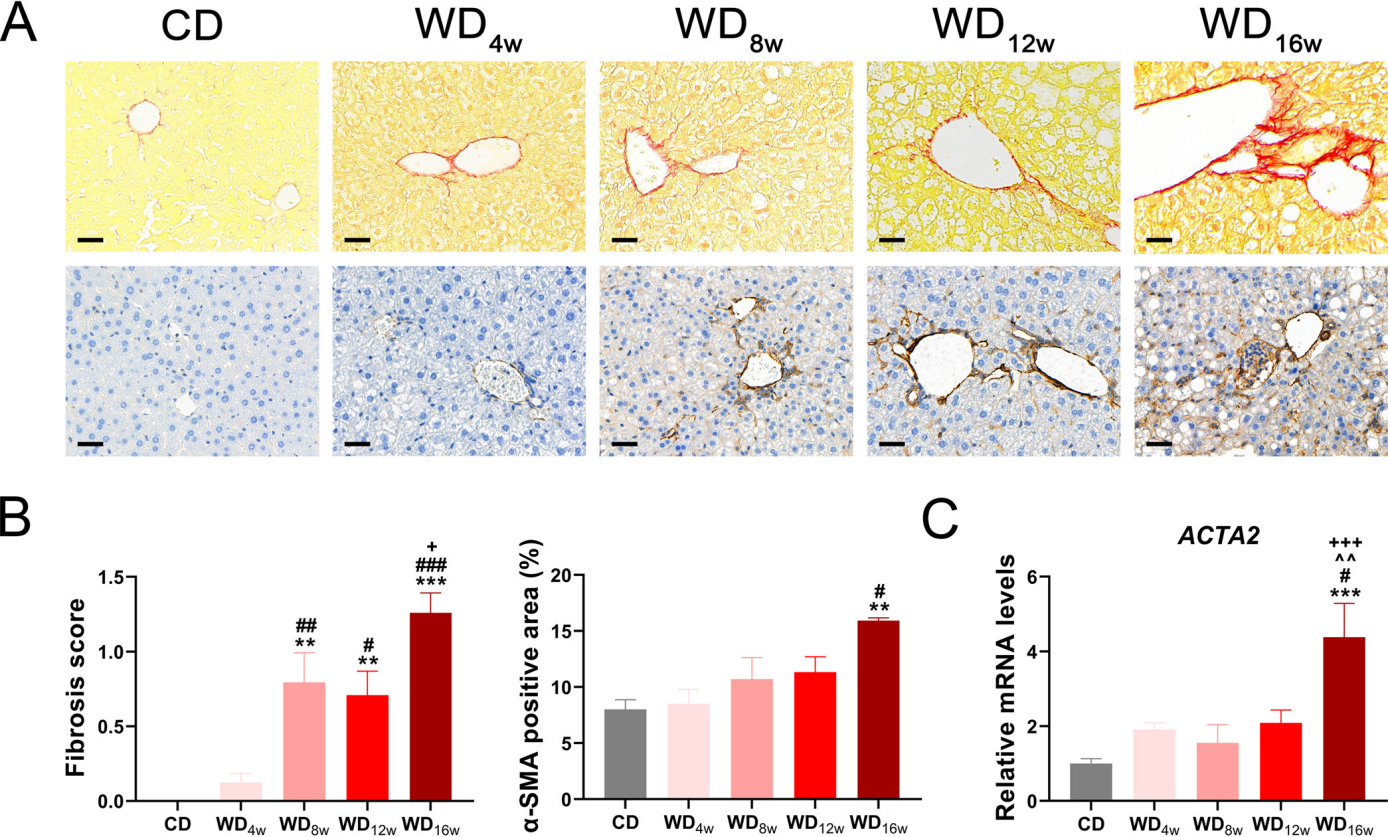
Longitudinal liver fibrosis progression in mice fed with a Western diet. (A) Representative images of the liver with Sirius red staining and *α-SMA* immunohistochemistry staining. Scale bar 20 μm. (B) Fibrosis score based on the Sirius Red staining (*n* = 8 to 9 per group) and *α-SMA* positive area (*n* = 6 per group). (C) Liver expression of *ACTA2* (*n* = 6 per group) at 0, 4th, 8th, 12th, and 16th week. Data are presented as mean ± SEM. * indicates a significant difference between the CD group and the other groups, **, *P* < 0.01; ***, *P* < 0.001; # indicates significant difference between the WD_4w_ group and the other groups, ^#^, *P* < 0.05; ^##^, *P* < 0.01; ^###^, *P* < 0.001; ^ indicates significant difference between the WD_8w_ group and the other groups, ^^^^, *P* < 0.01. + indicates significant difference between the WD_12w_ group and the other groups, ^+^, *P* < 0.05; ^+++^, *P* < 0.001.

Cirrhosis is a characteristic endpoint in clinical advanced NASH developing from liver fibrosis, which was driven by hepatocyte lipoapoptosis. Activated hepatic stellate cells, myofibroblasts, cholangiocytes, macrophages, and components of the pathological extracellular matrix act as fibrogenic effectors (19). We further investigated the degree of fibrosis in the liver. Sirius red and *α-SMA* immunohistochemistry staining indicated slight hepatic collagen deposition at the 8th and 12th weeks, and it developed into perisinusoidal or periportal fibrosis at the 16th week (Fig. 3A and B), which was consistent with the increased expression of profibrotic gene *ACTA2* (Fig. 3C).

**FIG 4:**
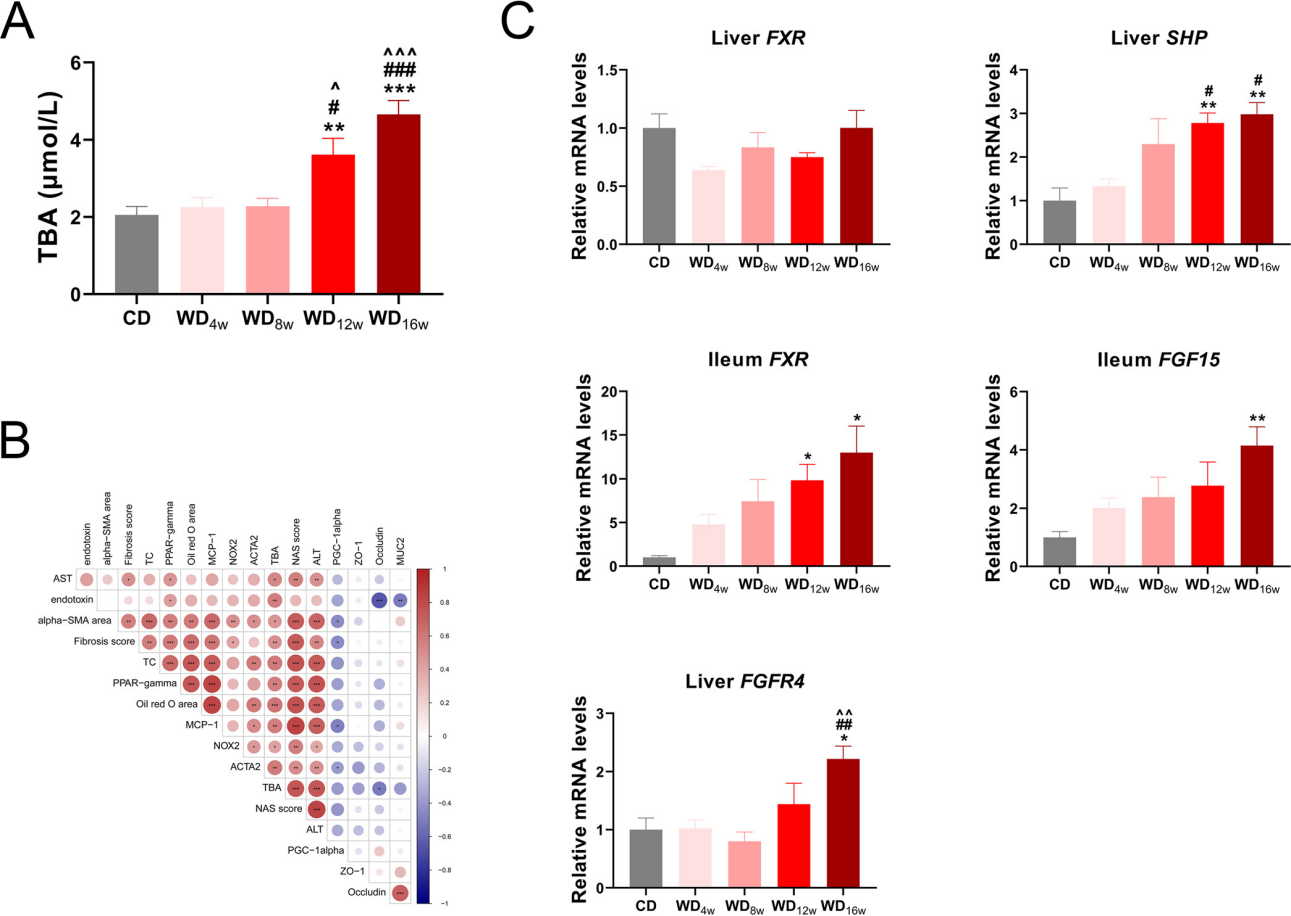
Longitudinal alternations in bile acid metabolism in mice fed with a Western diet. (A) Seral level of TBA (*n* = 8 per group), (B) Heatmap of Spearman’s rank correlation among TBA and NAFLD indexes. The color key indicates the strength of correlation (r value). Dark red indicates a more positive correlation, dark blue indicates a more negative correlation and white indicates no correlation. Correlations with *P* < 0.05 and *r *> 0.4 are regarded as related and significant. *, *P* < 0.05; **, *P* < 0.01; ***, *P* < 0.001. (C) Liver expressions of *FXR*, *SHP*, and *FGFR4* and ileum expressions of *FXR* and *FGF15* at 0, 4th, 8th, 12th, and 16th week (*n* = 6 per group). Data are presented as mean ± SEM. * indicates a significant difference between the CD group and the other groups, **, *P* < 0.01; ***, *P* < 0.001; # indicates significant difference between the WD_4w_ group and the other groups, ^#^, *P* < 0.05; ^##^, *P* < 0.01; ^###^, *P* < 0.001; ^ indicates significant difference between the WD_8w_ group and the other groups, ^^^^, *P* < 0.01. + indicates significant difference between the WD_12w_ group and the other groups, ^+^, *P* < 0.05; ^+++^, *P* < 0.001.

Bile acids play remarkable roles in the progression and treatment of NASH-associated liver fibrosis and circulating levels of bile acids alter dramatically from NAFL to NASH (20, 21). To explore longitudinal bile acid homeostasis in NAFLD progression, we conducted total bile acid (TBA) quantification and found seral TBA levels were elevated at the 12th and 16th weeks (Fig. 4A). Subsequent correlation analysis revealed relationships among TBA and NAFLD-associated indexes. Seral TBA level was highly positive associated with lipid metabolism indexes seral TC level, *PPAR-γ* expression, and Oil red O area (*r* = 0.58, *r* = 0.57, *r* = 0.61, respectively), seral ALT and AST levels (*r* = 0.74, *r* = 0.53, respectively), gut permeability indexes *Occludin* expression and serum endotoxin level (r = −0.52, *r* = 0.56, respectively), and fibrosis indexes *α-SMA* area, fibrosis score and *ACTA2* expression (*r* = 0.47, *r* = 0.50, *r* = 0.58, respectively). Further, indexes related to oxidative damage (expressions of *PGC-1α* and *NOX2*) presented a close interrelationship with fibrosis indexes. (Fig. 4B).

Farnesoid X receptor (FXR) is a bile acid receptor that regulates glucose and lipid metabolism and participates in fibrosis progression (20, 22). Therefore, we investigated expressions of FXR pathways in the liver and ileum and found liver *FXR*-*SHP* axis and ileum *FXR*-*FGF15*-*FGFR4* axis were both activated in the WD_12w_ and WD_16w_ group (Fig. 4C).

Together, these results support that abnormal lipid metabolism results in accumulated bile acids, which activate FXR pathways and promote the process of borderline NASH progressing to NASH (8th to 16th week), and oxidative damage occurs in this period.

### Western diet reshaped gut microbial community at multiple levels.

**FIG 5:**
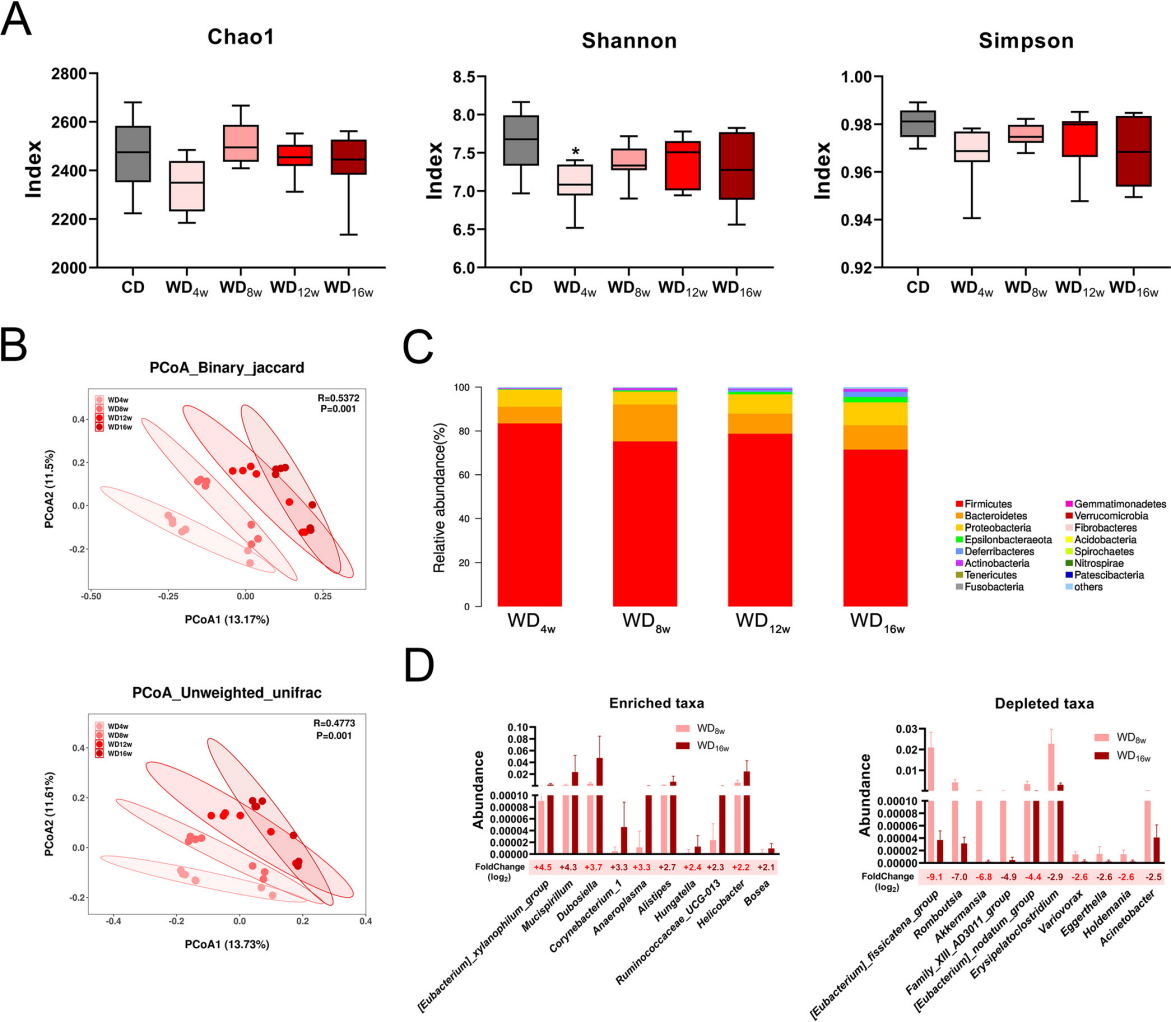
Longitudinal gut microbiota alterations in mice fed with a Western diet. (A) α-diversity indexes Chao1, Shannon, and Simpson at 0, 4th, 8th, 12th, and 16th week. (B) PCoA based on Binary_jaccard and Unweighted_unifrac among the WD_4w_, WD_8w_, WD_12w_, and WD_16w_ groups (*n* = 7 per group). (C) Relative abundance at the genus level in THE WD_4w_, WD_8w_, WD_12w_, and WD_16w_ groups (*n* = 7 per group). (D) Top 10 enriched and depleted taxa between the WD_8w_ and WD_16w_ groups (*n* = 7 per group). Data are presented as mean ± SEM. * indicates a significant difference between the CD group and the other groups, *, *P* < 0.05.

NAFLD progression is usually accompanied by gut microbiota alternations. To trace longitudinal microbial changes in the gut, we employed 16S rRNA sequencing to compare shifted gut microbiota along with the WD intervention. Thirty-five cecal content samples were collected at five time points of NAFLD (0, 4th, 8th, 12th, and 16th weeks; *n* = 7 each), and a total of 8207 OTUs were clustered. Microbial richness was initially decreased in the 4th week but showed a slight rebound trend afterward based on α-diversity indexes (Chao1, Shannon, and Simpson) (Fig. 5A). Additionally, β-diversity (principal coordinate analysis [PCoA] based on binary Jaccard and unweighted Unifrac) annotated that community compositions were constantly shifted in NAFLD progression (Fig. 5B). Subsequently, we quantified the relative abundance of microbial taxa at multiple levels to identify characteristic microbes of each stage. At the phylum level, the ratio of *Firmicutes*/*Bacteroidetes* was decreased while *Proteobacteria* was enriched (Fig. 5C and Fig. S3B). At the family level, the abundance of short-chain fatty acid (SCFA) producer *Ruminococcaceae* declined, while opportunistic pathogen *Desulfovibrinaceae* was enriched (Fig. S2A). At the genus level, WD administration significantly diminished *Bacteroides* and accumulated *Dubosiella* compared between the WD_8w_ and WD_16w_ groups (Fig. S2B).

To identify characterized microbes in each NAFLD stage, a linear discriminant analysis effect size (LEfSe) analysis was conducted. Based on linear discriminant analysis (LDA) score, *Clostridia*, *Clostridiales*, *Ruminococcaceae*, *[Eubacterium]_fissicatena_group*, *Erysipelatoclostridium*, and *Enterobacteriaceae* were concentrated in the early NASH microbiota (4th and 8th week), whereas NASH with fibrosis harbored higher abundance in genus *Dubosiella*, *Lachnoclostridium*, *Helicobacter*, *Alistipes*, *Bosea*, and *Campylobacteria* (Fig. S3A). Further, we traced the longitudinal abundance of these characterized taxa and found constantly depleted *Clostridia* and *Ruminococcaceae* and increased *Dubosiella*, *Bosea*, *Helicobacter*, *Alistipes*, *Campylobacteria*, *[Eubacterium]_xylanophilum_group*, and *Lachnoclostridium* as NASH progressed. *[Eubacterium]_fissicatena_group*, *Romboutsia*, *Erysipelatoclostridium*, and *Enterobacteriaceae* were initially enriched at the 8th week but conversely decreased afterward (Fig. S3B).

To further identify potential microbes which drove the borderline NASH to NASH with fibrosis, we filtered the top 10 most enriched and depleted taxa between the WD_8w_ and WD_16w_ groups. *[Eubacterium]_xylanophilum*, *Mucispirillum,* and *Dubosiella* were the most enriched, while *[Eubacterium]_fissicatena_group*, *Romboutsia*, and *Akkermansia* were substantially depleted (Fig. 5D).

These results indicate that compositions of gut microbiota are greatly influenced by WD administration, while each NAFLD stage corresponds to a specific microbial composition, which means involved microbes could be explored as an indicator for NAFLD progression.

### Relationships of gut microbiota with NAFLD indexes indicated the potential of Akkermansia muciniphila as a biomarker for NAFLD diagnosis.

**FIG 6:**
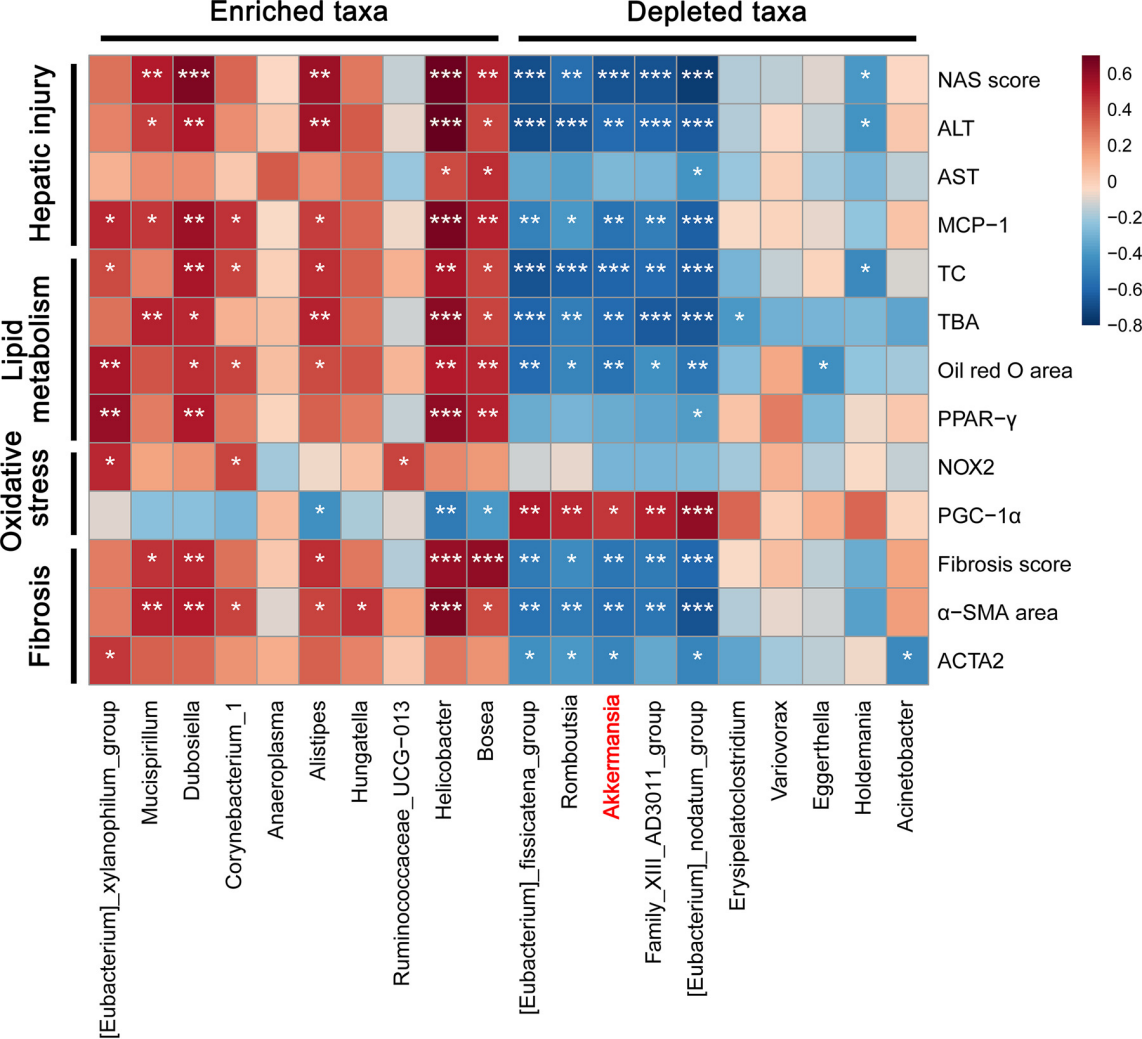
Relationships among altered gut microbes and NAFLD indexes in mice treated with a Western diet. Heatmap of Spearman’s rank correlation among top 20 most altered taxa and NAFLD indexes. The color key indicates the strength of correlation (r value). Dark red indicates a more positive correlation, dark blue indicates a more negative correlation and white indicates no correlation. Correlations with *P* < 0.05 and *r *> 0.4 are regarded as related and significant. *, *P* < 0.05; **, *P* < 0.01; ***, *P* < 0.001.

To further elucidate interrelationships between gut microbiota and NAFLD progression, we employed Spearman’s rank correlation analysis and uncovered several microbes closely associated with NAFLD features. As shown in Fig. 6, progressive NASH depleted taxa *[Eubacterium]_fissicatena_group*, *Family_XIII_AD3011_group*, *Romboutsia*, *Akkermansia*, and *[Eubacterium]_nodatum_group* exhibited overall negative correlations with hepatic injury, lipid metabolism, oxidative damage, and fibrosis. In contrast, progressive NASH enriched taxa *Mucispirillum*, *Dubosiella*, *Helicobacter*, and *Bosea* presented positive correlations with hepatic injury, lipid metabolism, and fibrosis, but showed no significant relationships with oxidative damage. Subsequently, we conducted correlation analysis to identify crosstalk between gut microbiota and bile acid metabolism and found *Alistipes*, *[Eubacterium]_fissicatena_group*, *Akkermansia*, and *[Eubacterium]_nodatum_group* play crucial roles in TBA accumulation. Liver *FXR*-*SHP* pathway was weakly related to the richness of *Dubosiella*, *Alistipes*, and *Helicobacter*, while NASH favored taxa *Mucispirillum* and the top 5 most depleted taxa exhibited strong relations with ileum *FXR*-*FGF15*-*FGFR4* pathway (Fig. S4).

**FIG 7:**
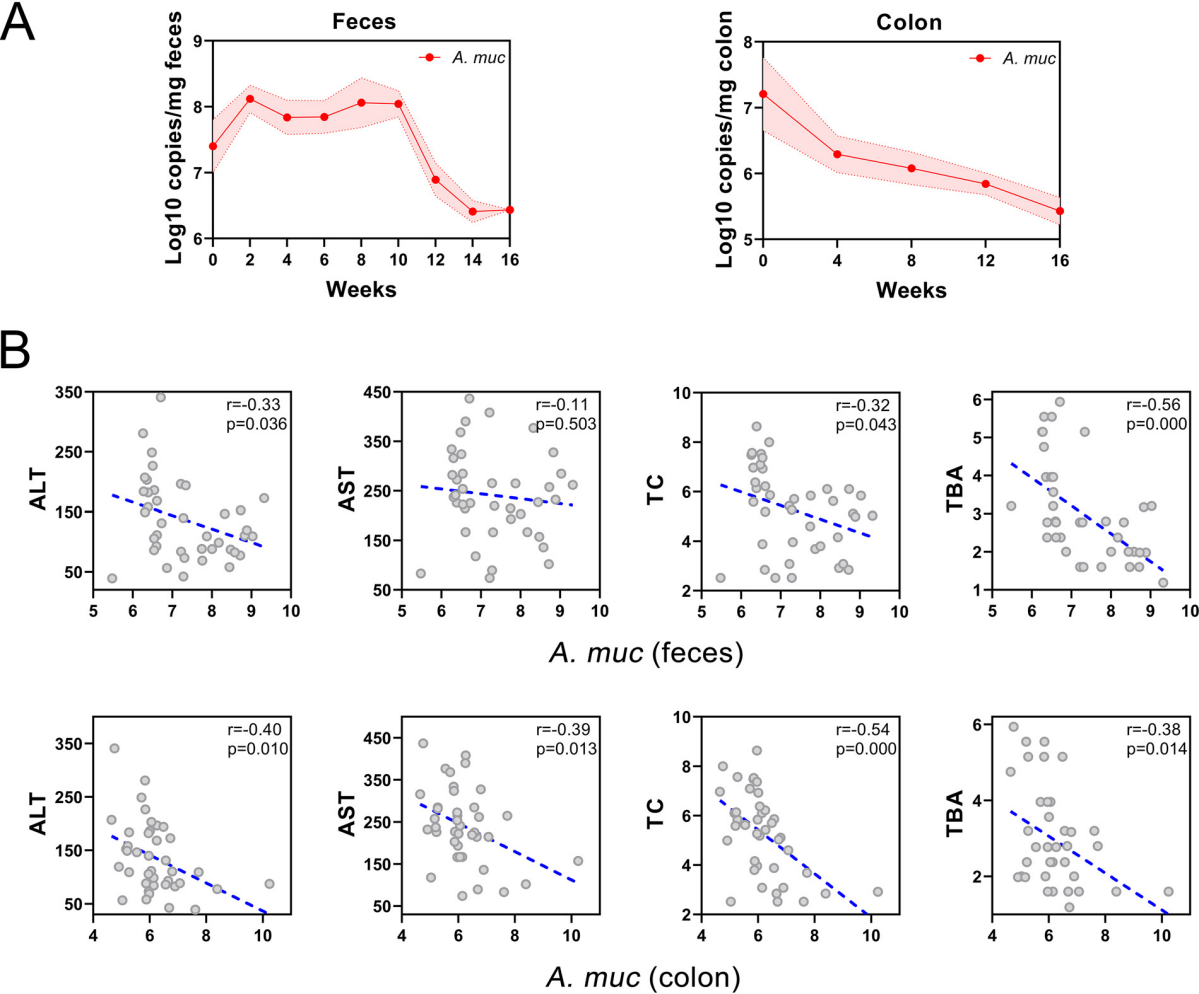
Relationship among longitudinal absolute abundance of A. muciniphila and NAFLD indexes in mice. (A) Absolute abundance of A. muciniphila in feces and colon mucosa at different time points. (B) Correlations among absolute abundance of A. muciniphila in feces and colon mucosa with ALT, AST, TC, and TBA. *A. muc*, A. muciniphila.

Akkermansia muciniphila is an abundant resident in the human intestinal tract and has exhibited its probiotic effects, including metabolic regulation, immune modulation, and gut barrier protection (23–25). Considering the strong correlations between the abundance of A. muciniphila, NAFLD indexes as well as bile acid metabolism, we conducted absolute qPCR quantifications focused on the longitudinal alternations of A. muciniphila in the feces and colon mucosa. As shown in Fig. 7A, the abundance of A. muciniphila in feces was initially increased in the first 10 weeks but was constantly decreased from the 12th week. In contrast, enrichment of A. muciniphila in colon mucosa was consistently depleted. Finally, we assessed its potential for clinical transformation and found the absolute abundances of A. muciniphila in feces and colon mucosa were exceedingly negative correlated with clinical markers ALT, AST, TC, and TBA (Fig. 7B). These results indicate that A. muciniphila has clinical potential for noninvasive diagnosis of NASH, although it exhibits little diagnostic efficiency in the early stage of NAFLD.

### Microbiota-derived predictive models exhibited great diagnostic accuracy to identify NAFLD and NASH.

**FIG 8:**
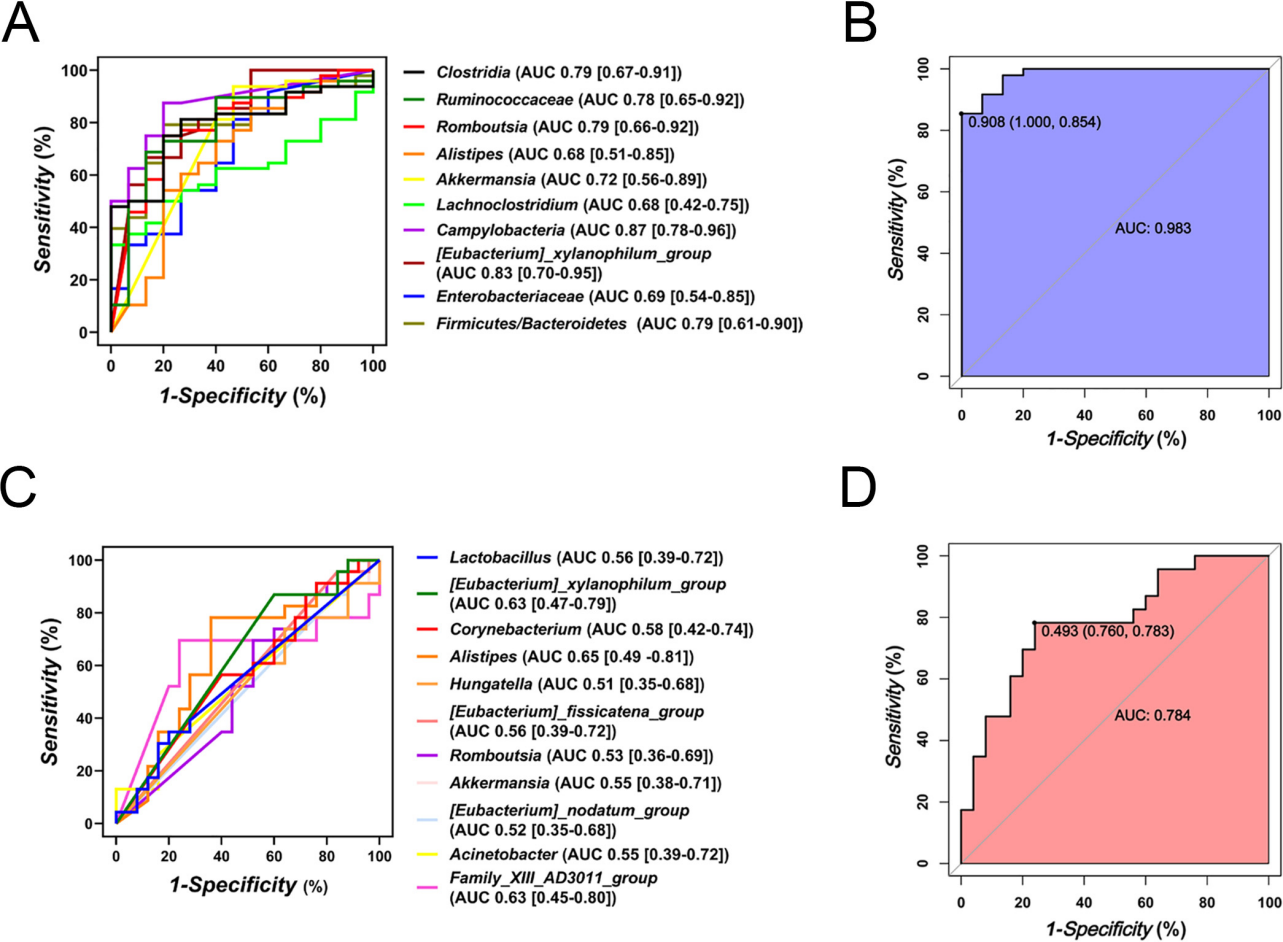
Diagnostic potential of microbiota-derived signatures in validation sets. ROC analysis of filtered taxa for NAFLD (A) and NASH (C) diagnosis. Performance of compound microbiota-derived ROC model built based on the filtered taxa for NAFLD (B) and NASH (D) diagnosis. AUC, area under the curve.

To further verify the potential efficiency of these filtered taxa, we assess the diagnostic accuracy using receiver operating characteristic curve (ROC) analysis on an American NAFLD cohort (Fig. 8). *Clostridia*, *Ruminococcaceae*, *Romboutsia*, *Akkermansia*, *Lachnoclostridium*, *Campylobacteria*, *[Eubacterium]_xylanophilum_group* and F/B ratio exhibited considerable efficiency to identify NAFLD (NAFL and NASH) from health controls (AUC > 0.75) (Fig. 8a). And compound microbiota-derived ROC model yielded an AUC of 0.983 (Fig. 8b). BA-related taxa *[Eubacterium]_xylanophilum_group, [Eubacterium]_xylanophilum_group* and Family_XIII_AD3011_group presented acceptable efficiency to distinguish NASH from NAFL (Fig. 8c), and the integrated model produced an AUC of 0.784 (Fig. 8d).

## DISCUSSION

NAFLD is a prevalent and progressive disease spectrum comprised of pathological ectopic fat accumulation, deranged adipokine, insulin resistance, increased oxidative damage, inflammation, and fibrosis) (8). However, the stage of NAFLD at which each parallel histological alternation occurs and the intercorrelations are confusing. Additionally, different types of lipids with different cytotoxic potential accumulated in the NAFL and distinctive reactions to lipotoxicity will lead to patient heterogeneity (26). Thus, it is essential to conduct NAFLD stratification based on its unique clinical profiles (27). Our study indicates that clinical NAFLD profiles occurred non-simultaneously in the experimental murine model. In detail, apparent lipid accumulation and inflammation occurred in the 8th week, while fibrosis together with oxidative damage appeared in the 16th week, in which impaired bile acid metabolism plays a considerable role. Mice after 16-week-feeding with WD presented severe hepatic lipid accumulation, inflammation, oxidative damage, insulin resistance, and fibrosis, all of which were consistent with NASH clinical profiles.

The primary driver of hepatic fibrosis in humans has been widely acknowledged as the activation of hepatic stellate cells (HSCs) (28, 29). Various factors contribute to HSCs activation, among which damaged hepatocytes-derived reactive oxygen species (ROS) can provide paracrine activation signals to HSCs (30). In this process, NADPH oxidase (NOX) serves as a transmembrane enzyme complex that produces ROS in response to a range of stimuli (31). What’s more, bile acids can stimulate the generation of mitochondrial-derived oxygen free radicals and promote their release from neutrophils and macrophages to cause oxidative damage (32). Thus, we concluded that this murine NAFLD model begins with simple steatosis, then lipid accumulated in the liver contributes to inflammation and bile acid dysbiosis, which subsequently results in oxidative damage, and finally, fibrosis occurs driven by these factors simultaneously.

Bile acids are products derived from cholesterol in lipid metabolism, which regulate glucose homeostasis, lipid and lipoprotein metabolism, energy expenditure, intestinal motility, bacterial growth, inflammation, liver regeneration, and hepatocarcinogenesis (22). Due to their lipotoxicity, regulations of lipid and carbohydrate metabolic pathways, and energy homeostasis, the balance of the bile acid pool is essential in NAFLD progression (20, 33). Intestinal crosstalk exists between gut microbiota and bile acid metabolism, as conjugated bile acids are uncoupled by bile salt hydrolase (BSH) related microbes and bile acids in turn modulate gut microbial composition by activating innate immunity in the small intestine (34, 35). In this study, we found that the seral TBA level was elevated and the FXR pathway in the liver and ileum were both activated in the NASH mice. Meanwhile, we found that impaired intestinal barrier function and elevated bacterial translocation in NASH development were associated with bile acid metabolism. And aggravated intestinal permeability will induce bacterial translocation to the liver (36). These findings indicated the possible alternations in the gut microbiota and cross talk in the gut-liver axis.

Therefore, we investigated longitudinal alternations in the gut microbiota, and observed constant shifts in microbial compositions with long-term WD intervention, while microbial richness does not significantly change. Meanwhile, we found different microbial profiles in correspondence to different NAFLD stages. Gut microbiota in NAFL with simple steatosis (4th week) is characterized by the highest abundance of *Clostridia* and *Ruminococcaceae*, both of which were decreased with NAFLD progressing. *Clostridia* consists of major BSH-producing bacteria, such as *Clostridium* and *Eubacterium*, which are vital in the enterohepatic circulation of bile acids (37). *Ruminococcaceae* can produce SCFAs to the protection of gut barrier and maintain intestinal immune homeostasis (38, 39). This evidence may explain why gut barrier leakage and bile acid dysbiosis usually do not occur in the early NAFL but deteriorate in further progression.

Further, the microbial features of borderline NASH are targeted as *[Eubacterium]_fissicatena_group*, *Romboutsia*, *Erysipelatoclostridium*, and *Enterobacteriaceae*, which are associated with obesity-related metabolic disorders and intestinal inflammation (40–42). These data demonstrate that gut injury derived from accumulated lipid may participate in NASH progression.

Finally, microbial biomarkers of NASH with fibrosis are defined as oxidative damage-related microbes *Dubosiella*, *Bosea*, and *Helicobacter*, and bile-tolerant microorganisms *Alistipes*. *Dubosiella* and *Bosea* were found enriched in intestinal injury with altered expressions of antioxidant genes (43, 44), while Helicobacter pylori are linked to chronic inflammation-related oxidative damage and DNA damage through its virulence factors, including cytotoxin associated gene A (*cagA*), vacuolating cytotoxin A (*vacA*), γ-glutamyl transferase (GGT), urease and neutrophil-activating protein A (NapA) (45). *Alistipes* were found bile-resistant and related to gut dysbiosis in NAFLD and liver fibrosis (46, 47). These results present that the intestinal microbial composition of NASH tends to be tolerant to excessive bile acids and ROS in the gut, indicating the situation of oxidative damage and bile acid accumulation that the liver faces.

There is no effective treatment for NASH so far, thus early diagnosis is essential for the prognosis of NASH. A liver biopsy specimen is the only accepted method to differentiate NASH from simple steatosis, even though it usually causes complications such as bleeding, infection, bile leak, damage to other organs, and rare mortality risk (<0.01%). Sampling bias, underestimation of disease severity, and interobserver variability will attenuate its accuracy (48, 49). Various noninvasive diagnosis tools of NAFLD based on blood biomarkers, imaging biomarkers, and non-proprietary biomarkers have been developed, but diagnostic efficiency is poorly satisfactory (50). However, the development of omics technologies provides alternatives to identify novel biomarkers for NAFLD, NASH, and fibrosis, such as lipidomics, proteomics, metabolomics, and microbiomics (49). Considering longitudinal alternations of gut microbiota in NAFLD progression, microbial signals detected from easily and repeatedly accessible feces samples have the potential to be explored as dynamic indicators for NAFLD noninvasive diagnosis.

A. muciniphila is a mucin-degrading bacteria and has been speculated to strengthen metabolic regulation, anti-inflammatory activity, and anti-cancer immunosurveillance by producing acetate and propionate to restore gut barrier (23, 51–54). A. muciniphila mostly colonizes in the intestinal mucosal layer, with the largest numbers in the cecum and colon (24, 55). Recent research demonstrated that A. muciniphila improves lipid oxidation and gut-liver interaction by modulating l-aspartate metabolism, which provided new horizons in clinical applications of A. muciniphila for NAFLD (56). Further, HSCs activation was found to be inhibited by live and pasteurized A. muciniphila and its extracellular vehicles (EVs) in lipopolysaccharide (LPS)-activated LX-2 cells and high-fat diet and carbon tetrachloride (CCl4) treated mice, which exhibits the anti-fibrosis capability of A. muciniphila (57, 58). In this study, we found an abundance of A. muciniphila in colon mucosa was steadily declined with NAFLD developing, while feces richness showed a “n”-shaped alternation, and both showed strong negative relationships with clinical NAFLD indexes. These phenomena indicate the mucosal colonization of A. muciniphila is impaired in NAFLD progression. Dietary fiber deprived WD may degrade the colonic mucus layer, consume available ingredients for the metabolism of A. muciniphila and ultimately influence its colonization (59). Because the microbial sample of colonic mucosal is difficult to acquire unless, through invasive colonoscopy biopsy specimen (60), feces sample is more attainable and should be taken into consideration. Whereas initial fecal abundances of A. muciniphila alter unstably, it exhibits diagnostic potential at the stage from borderline NASH progressed to NASH and late fibrosis. These data suggest that A. muciniphila has the potential to be adopted as a specific microbial indicator for NASH progression.

Importantly, these microbiota-derived signatures achieved great diagnostic accuracies in the American validation cohort. we demonstrated that a diagnostic model based on the mouse core gut microbiome could identify human early NAFL and advanced NASH as well. Previously, Oh et al. (17) established a microbiota-derived model to identify fibrosis stages in NASH patients, including Veillonella parvula, Veillonella atypica, Ruminococcus gnavus, Clostridium bolteae, *Acidaminococcus* sp. *D2*, Eubacterium eligens, Eubacterium rectale, and Faecalibacterium prausnitzii (17). Notably, they combined microbiome signature and clinical profiles (BMI, albumin, ALT, AST, bilirubin, and cholesterol, for example) and acquired higher diagnostic accuracy (17). Our study primarily focused on the identification of NAFLD from healthy cohorts and NASH from NAFLD patients, which is also of great clinical significance. Because BA-related gut microbes exhibited great accuracy to distinguish NASH from NAFL in our study, which are in accordance with the impaired BA metabolism in NASH, thus we further infer that gene expressions of BA pathways and serum BA level also have great potential in NASH diagnosis, suggesting a combination of gut microbiome signature and BA profiles could be an approaching tool for noninvasive diagnosis of NASH.

Several limitations exist in the present study. First, discovered microorganisms are not located at the species level due to the limited sequence of length, which will influence the accuracy of diagnosis. More specific species indicators based on metagenome sequencing should be employed, but the cost is a concern for clinical diagnosis. Second, we did not take batch and cage effects and diurnal differences into consideration, and the sample size limit may bring errors. Third, the limited access to clinical NAFLD indexes in the American cohort results makes it infeasible to integrate the gut microbiome with clinical indicators for diagnosis, which may higher diagnostic accuracy.

In conclusion, we detected several parallel alternations that do not occur simultaneously in the murine NAFLD model fed with WD that begins with simple steatosis, develops into hepatic inflammation, and subsequently causes oxidative damage and fibrosis under bile acids accumulation. Further, we concluded three different microbial compositions in NAFLD development, which correspond to specific histological changes and may function in the auxiliary diagnosis of NASH. In addition, we established two microbiota-derived diagnostic models for NAFLD and NASH, which exhibit valuable diagnostic potential. Our finding of core gut microbiota-derived signatures lays the foundations for a stool-based and noninvasive diagnostic tool for NASH among NAFL individuals.

## MATERIALS AND METHODS

### Mice and sample collection.

C57BL/6J male mice (6 weeks of age) from Shanghai SLAC Laboratory Animal, Co., Ltd. were housed in a specific pathogen-free environment at 20 to 22°C with a set 12-h-to-12-h light-dark cycle. To establish the murine NAFLD model, mice were fed with a Western diet (WD group, protein, 20%; fat 60%, carbohydrates, 20%) after 1 week of adaptation and subjected to high glucose/fructose (18.9 g glucose and 23.1 g fructose in 1 L of tap water) in drinking water for 0, 4, 8, 12, 16 weeks, respectively. During the experiments, body weight, BMI, and food intake were assayed weekly, and feces of mice in the WD_16w_ group were collected in a sterile container twice a week. The animals were anesthetized and sacrificed at 4th, 8th, 12th, and 16th week time points to harvest liver, ileum, colon, blood, and feces for further analysis. Mice sacrificed in the 0^th^ week were set as the control group (CD group).

### Serum assays.

Seral concentrations of alanine aminotransferase (ALT), aspartate aminotransferase (AST), triglyceride (TC), total cholesterol (TG), and total bile acid (TBA) were measured using the Hitachi 7600-210 automatic analyzer (Hitachi, Tokyo, Japan).

### Histopathology analysis.

Liver tissue was prefixed in 10% paraformaldehyde for 24 h, embedded in paraffin and sectioned in 2 μm-thickness, and stained with hematoxylin and eosin (H&E) and Sirius red. To estimate the degree of NAFLD progression, we applied a NAFLD activity score (NAS) system containing steatosis, lobular inflammation, and hepatocellular ballooning (score 0 to 2: non-NASH; 3 to 4: borderline NASH; 5 to 8: NASH) (61). The extent of liver fibrosis was further assessed based on Sirius red staining as previously reported.

Frozen liver sections in 4 μm-thickness were fixed in 4% neutralized formaldehyde and stained with Oil red O, and the area of the fat droplet was counted to evaluate fat accumulation in the liver.

### Immunofluorescence staining.

Frozen liver sections were stained with dihydroethidium (DHE) to assess reactive oxygen species (ROS) levels in the liver.

Terminal ileum sections were dewaxed, rehydrated, and treated with 3% H202. Specimens were primarily incubated with ZO-1 primary antibody (1:500, Proteintech, LA, USA) overnight at 4°C and stained with fluorescein isothiocyanate (FITC)-conjugated goat anti-rabbit (1:500, Beyotime, Shanghai, China) at room temperature for 1 h. After washing twice with PBS, sections were mounted with a mounting medium (Beyotime) and observed through a fluorescence microscope (Eclipse 80i; Nikon, Tokyo, Japan).

### Immunohistochemistry staining.

Paraffin-embedded liver sections were incubated with an α-SMA antibody (Abcam, Cambridge, UK) and followed by incubation with an HRP-conjugated secondary antibody (Abcam). The positive area of *α-SMA* staining was assessed using the ImageJ IHC profiler.

### RNA extraction and quantitative real-time PCR.

Total liver and ileum RNA was extracted by an RNeasy Pro kit (Qiagen) according to the manufacturer’s protocols. The relative mRNA expression was assessed by the VIIA7 real-time PCR system (Applied Biosystems, CA, USA) and normalized to the levels of the housekeeping gene *GAPDH*. The primer sequences are listed in Table S1.

### Intraperitoneal glucose tolerance test (IGTT) and serum insulin quantification.

IGTT was conducted on the 24^th^ week as previously described. Briefly, mice in the WD_16w_ group were fasted for 16 h and then administered with 2 g/kg glucose by intraperitoneal injection. Blood glucose levels were evaluated at 0, 15, 30, 60, 90, and 120 min after injection with a glucometer (Roche, Basel, Switzerland). The area under the curve (AUC) was calculated by GraphPad. Serum insulin concentrations were detected using an ELISA kit (Abcam).

### Serum endotoxin assays.

Bacterial translocation biomarker endotoxin was quantified by a commercial ELISA kit in extracted serum according to instructions.

### 16s rRNA sequencing.

Total bacterial DNA was extracted from cecal contents using a DNeasy PowerSoil Pro kit (Qiagen, CA, USA) and its concentration and integrity were verified by NanoDrop (Thermo Fisher Scientific, MA, USA) and agarose gel electrophoresis. PCR amplification of the V3 to V4 variable regions of 16S rRNA genes was then carried out with universal primers (343F: 5′-TACGGRAGGCAGCAG-3′; 798R: 5′-AGGGTATCTAATCCT-3′). After amplification, purification, and qualification, sequencing data were performed on an Illumina NovaSeq6000 platform (Illumina Inc., CA, USA) to construct the library.

Sequencing reads were trimmed by Trimmomatic software, assembled with FLASH software, and clustered to operational taxonomic units (OTUs) using VSEARCH software with a 97% similarity cutoff (62–64). Finally, the QIIME package was employed to select representative read. The α-diversity based on Chao1, Shannon, and Simpson index was used to evaluate microbial diversity, the PCoA based on Binary_jaccard and Unweighted_unifrac distance were conducted to assess microbial community alternations, and linear discriminant analysis effect size (LefSe) coupled with LDA was used to identify characteristic taxa in correspondence to each NAFLD stage (65).

### DNA extraction and absolute qPCR quantification.

Total feces and colon DNA was extracted using a DNeasy PowerSoil Pro kit (Qiagen) following the instructions. To validate the absolute abundance of A. muciniphila in the 16S rRNA sequencing results, we conducted absolute qPCR quantification as previously described. In brief, a plasmid containing the A. muciniphila 16S rRNA gene (Tsingke Biotechnology Co., Ltd., Beijing, China) was used for drawing a standard curve, and absolute abundance was calculated and shown as 16S copies per milligram feces.

### Validation cohort and microbiota-derived predictive model.

Two microbiota-derived predictive models for NAFLD and NASH based on 10 and 11 characteristic taxa were verified in an American proband cohort, including 15 non-NAFLD control, 25 NAFL, and 23 NASH patients. To compute and visualize AUC from the ROC outcome, the pROC package was utilized.

Sequence data of the external American cohort were acquired from MG-rast (available at: http://metagenomics.anl.gov/linkin.cgi?project=1195).

### Statistical analysis.

One-way ANOVA followed by Tukey’s test was used to analyze differences among groups showing homogeneity of variance, otherwise, Brown-Forsythe ANOVA followed by Games-Howell’s test was applied. Relationships among gut microbiota and NAFLD indexes were assayed using Spearman’s rank correlation analysis. All data were presented as mean ± SEM, and a *P* < 0.05 was defined as statistically significant. GraphPad Prism 6 (GraphPad Software Inc., CA, USA) and ImageJ (Rawak Software Inc., Stuttgart, Germany) were used to analyze data and draw figures.

### Data availability.

The 16S rRNA sequencing data sets have been uploaded to the Sequence Read Archive (SRA) database under BioProject number PRJNA787736.
